# fNIRS Optodes’ Location Decider (fOLD): a toolbox for probe arrangement guided by brain regions-of-interest

**DOI:** 10.1038/s41598-018-21716-z

**Published:** 2018-02-20

**Authors:** Guilherme Augusto Zimeo Morais, Joana Bisol Balardin, João Ricardo Sato

**Affiliations:** 1 NIRx Medizintechnik GmbH, Gustav-Meyer-Allee 25, 13355 Berlin, Germany; 2 Instituto do Cérebro, Hospital Israelita Albert Einstein, 05652-900 São Paulo, Brazil; 3 Center for Mathematics Computing and Cognition, Universidade Federal do ABC, 09210-180 São Bernardo do Campo, Brazil

## Abstract

The employment of functional near-infrared spectroscopy (fNIRS) as a method of brain imaging has increased over the last few years due to its portability, low-cost and robustness to subject movement. Experiments with fNIRS are designed in the face of a limited number of sources and detectors (optodes) to be positioned on selected portion(s) of the scalp. The optodes locations represent an expectation of assessing cortical regions relevant to the experiment’s hypothesis. However, this translation process remains a challenge for fNIRS experimental design. In the present study, we propose an approach that automatically decides the location of fNIRS optodes from a set of predefined positions with the aim of maximizing the anatomical specificity to brain regions-of-interest. The implemented method is based on photon transport simulations on two head atlases. The results are compiled into the publicly available “fNIRS Optodes’ Location Decider” (fOLD). This toolbox is a first-order approach to bring the achieved advancements of parcellation methods and meta-analyses from functional magnetic resonance imaging to more precisely guide the selection of optode positions for fNIRS experiments.

## Introduction

Functional near-infrared spectroscopy (fNIRS) is a technique capable of measuring concentration changes of oxygenated and deoxygenated hemoglobin from the variations of absorbed near-infrared light during its transportation in tissues^1^. The employment of fNIRS to assess brain activity has increased over the last few years due to its advantages over functional magnetic resonance imaging (fMRI) and electroencephalography (EEG) mainly concerning its robustness to artifacts due to motion^2^, thus enabling a greater gamut of naturalistic experiments^3–8^.

**Figure 1:**
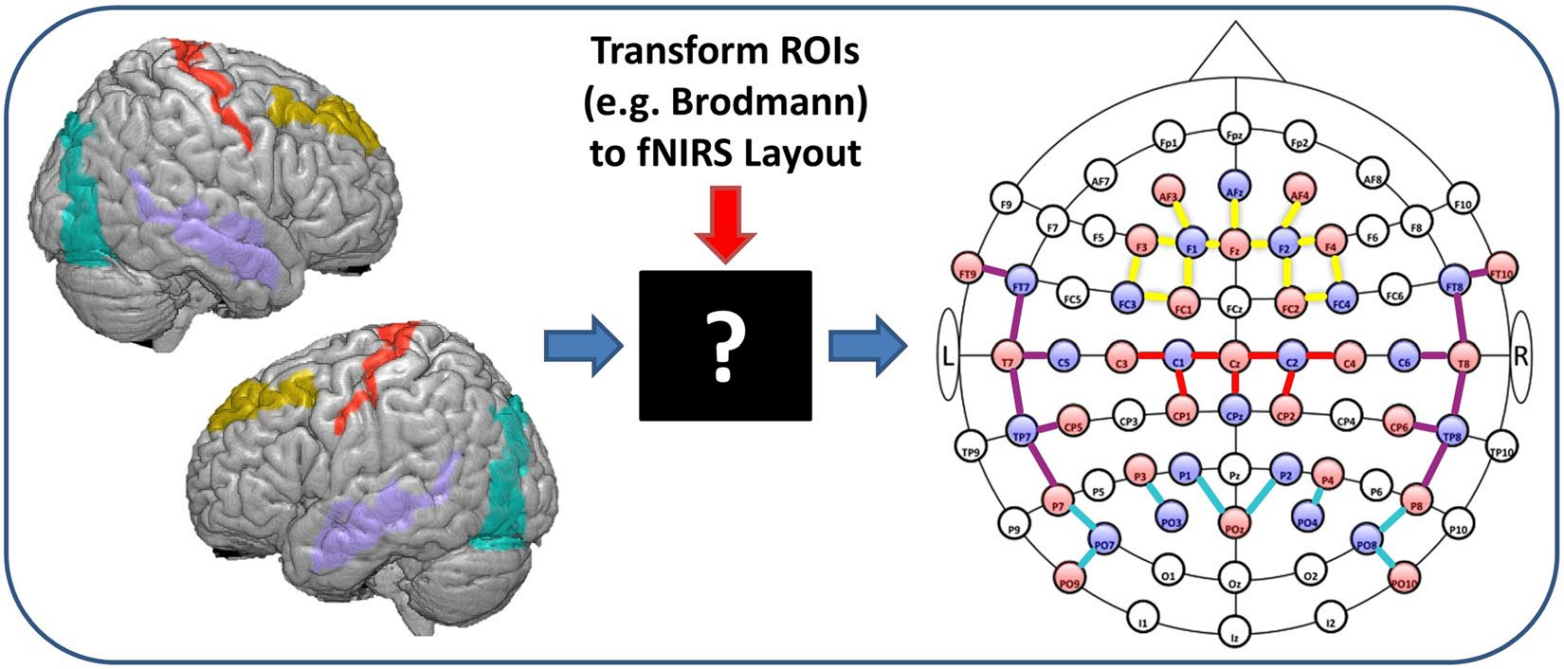
Challenge commonly faced when designing an fNIRS experiment to assess a set of regions of interest expected to be activated according to a study hypothesis: the translation to an fNIRS optode layout by choosing appropriate sources and detectors positions to maximize anatomical specificity to regions of interest. Illustrated are Brodmann areas 4, 9 and 19 and 21 and the fNIRS cap layout with corresponding color-coded channels.

While fMRI is capable of measuring whole-brain functional activity along with the structural image of the individual, fNIRS experiments are designed with a limited number of sources and detectors (optodes). The optodes are positioned on selected portion(s) of the scalp with an expectation to assess the activity of a set of brain cortical regions that are relevant to the designed experiment. However, the translation of regions of interest to the placement of optodes on a measuring cap (shown in Fig. 1) remains a challenge for experimental design with fNIRS.

A few recent studies have suggested approaches to overcome this challenge, e.g. for epileptic discharges^9^, or based on iterative probe geometry modifications^10^, which was extended to the voxel-space for an image-based approach^11^. Herein, we propose an alternative approach to automatically decide optodes positions based on 10–10 and 10–5 systems^12^ according to a set of brain regions of interest. This method is based on the sensitivity profile from photon transport simulations run on two head atlases (Methods section). The results were compiled into a toolbox to facilitate the definition of optodes positions, the fNIRS Optodes’ Location Decider (fOLD).

## Methods

### Tissue segmentation

As different tissues of the human head present different optical properties (e.g. absorption and scattering)^13,14^, it is necessary to segment the atlases to be used for photon transport simulations. The segmentation results in discrimination of five tissues: scalp, skull, cerebrospinal fluid (CSF), and gray and white matter.

The segmentation algorithm implemented in SPM12^15^ with default parameters has been used to segment the T1 image of the Colin27 head atlas^16^ provided within the MRIcron software^17^ as “ch2.nii.gz”.

Briefly, the segmentation procedure in SPM12 returns probability maps for each of the five tissues of our interest. For each tissue, a NIfTI file is generated with an image with same size and origin as in the input file (e.g. Colin27 atlas) and each voxel is set to the probability of being part of a given tissue. And the sum of the probabilities of a given voxel along head tissues plus air is 1. For example, voxel (x = 57, y = 126, z = 144) of Colin27 atlas corresponding to MNI coordinate (x = −34, y = 0, z = 72) presented probability 86.27% for skull and 13.73% for CSF.

To create a single image file for the final tissue segmentation, we have defined each voxel to be part of a given tissue if its probability was higher than all other tissues and if it was greater than 0.2. The latter was particularly important for boundary voxels e.g. between scalp and air. Voxels corresponding to scalp were assigned the value 1, skull 2, CSF 3, gray matter 4 and white matter 5. All voxels that did not fulfill the comparisons for any of tissue was assigned value 0 (air).

**Figure 2:**
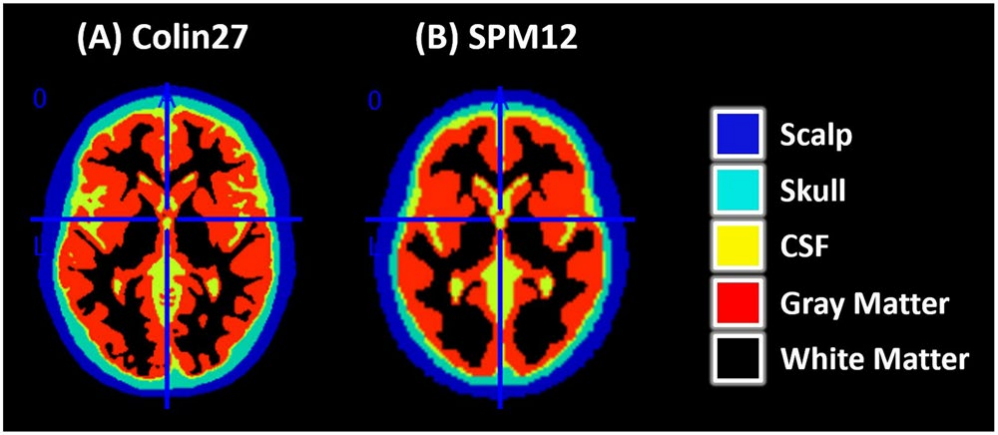
Axial view of the tissue segmentation of (**A**) Colin27 template and (**B**) SPM12 tissue probability maps (TPM.nii). This resulted on five layers: scalp (blue), skull (cyan), cerebrospinal fluid (CSF, yellow), gray matter (red) and white matter (black).

As this procedure could potentially result in unitary holes inside the tissue (i.e. tissue voxels assigned to air), the resulting image was smoothed in SPM12 with full width at half maximum (FWHM) of [2 mm, 2 mm, 2 mm]. The values in the smoothed image were rounded in Matlab2017a. The resulting segmentation of Colin27 is depicted in Fig. 2A.

In addition to the Colin27 head model, which is based on 27 averages of a single subject, we have considered a second head atlas based on the tissue probability maps provided in SPM12. These maps have been generated based on 549 subjects from the IXI dataset^18^. The specific subjects used to compute TPM.nii can be found in spm_templates.man^19^. We spatially resampled^20^ the TPM.nii image from 1.5 × 1.5 × 1.5 mm^3^ to 2 × 2 × 2 mm^3^ and followed the same procedure as for Colin27 to obtain the segmented image (herein called “SPM12”), illustrated in Fig. 2B.

### Optodes coordinates

Once the tissue segmentation procedure was performed, the scalp boundaries were defined for both brain atlases of interest, and thus we proceeded with the spatial localization of optodes to be used for the photon migration simulations.

We initially considered the 10–10 international system as primary reference for optodes placement as its positions can be identified from a set of fiducial points (e.g. nasion, inion and left/right preauricular points)^12^, which may be visually determined in head models.

**Figure 3:**
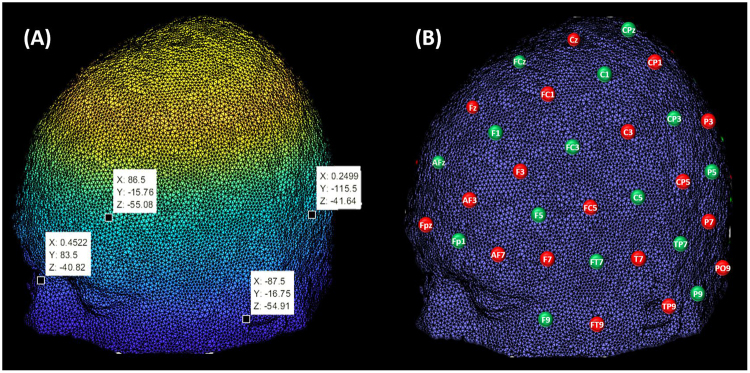
(**A**) MNI space localization (in mm) of fiducial points on mesh of Colin27. (**B**) Left view of sources (red) and detectors (green) positions on the 10–10 international system that have been initially considered for photon transport simulation (Methods section).

First, we converted the image file of the tissue segmentation into mesh by using the function ‘v2s’ provided in the iso2mesh toolbox^21,22^. We also corrected the coordinates to MNI space by accounting for the voxel size and origin of the image. After plotting the corrected mesh in Matlab2017a, we visually identified four fiducial points (depicted in Fig. 3A) whose spatial identification followed the definition provided by Jurcak *et al*.^12^ for nasion, inion and preauricular points.

After the fiducial points have been located, we used the functions provided within the tool Mesh2EEG^23,24^, which returned as output the MNI coordinates of 329 positions of the 10–5 international system. From these, we initially considered 74 positions within the 10–10 system, as illustrated in Fig. 3B.

The assignment of each position to a source or a detector was empirically done with the goal to maximize the number of channels, while also achieving a similar number of resulting sources and detectors. We proceeded by alternating sources and detectors on neighboring positions, which resulted on 38 sources and 36 detectors.

### Photon transport simulation

To assess the migration of photons within the head tissues and identify the areas of the brain that can potentially be measured by each measuring channel (i.e. a source-detector pair), we have performed simulations of photon transport from each optode position with Monte Carlo Extreme (MCX, v2017.3), a software that accelerates simulations with Graphics Processing Units, as described in refs^25,26^.

As input for MCX, we provided an *.inp text file with relevant information for the simulation, as exemplified in ref.^25^. We defined the number of photons to be launched to 10^8^. The optode position has been set in coordinates in voxel space. The original direction of the photon was placed towards the center of the image, which we defined as voxel coordinates corresponding to the origin (0, 0, 0) in MNI space. The binary volume file (*.bin) has been created from the segmented NIfTI file of interest (Colin27 or SPM12). Both time gate’s step and end were set to 5 ns to result on a single flux distribution. Voxel size and dimensions were defined according to the atlas of interest. Optodes were modelled as pencil beam (default setting in MCX). Finally we considered five different media (tissues described in Methods section), whose optical properties were set in accordance to Strangman *et al*.^27^ (Table 1).

**Table 1:** Optical properties provided as input for Monte Carlo simulations.

Tissue	Scattering (1/mm)	Anisotropy	Absorption (1/mm)	Refraction
Scalp	0.72	0.01	0.017275	1
Skull	0.92	0.01	0.011925	1
CSF	0.01	0.01	0.002500	1
Gray	1.10	0.01	0.019500	1
White	1.35	0.01	0.016900	1

For each optode simulation, the MCX binary was called along with the following settings: (i) enable automatic thread and block configuration to maximize speed (-A); (ii) disable the solution normalization (-U 0); (iii) save the flux field (-S 1); and (iv) divide photons into 100 groups (-r 100). The latter was required to avoid “kernel launch time-out” error due to non-dedicated graphics card^28^. Simulations were performed in an Ubuntu 16.04.02 LTS (Xenial Xerus) with Intel Xeon E5 2650 v3 2.3 GHz, GeForce Gtx 770 and CUDA 8.0.

The flux field solution of each optode simulation was normalized as described in Boas *et al*.^29^ and following its implementation in AtlasViewer^30^ by dividing the output by the number of simulated photons and correcting the resulting photon fluence to respect energy conservation (all photons launched should either exit the media or be absorbed by it).

**Figure 4:**
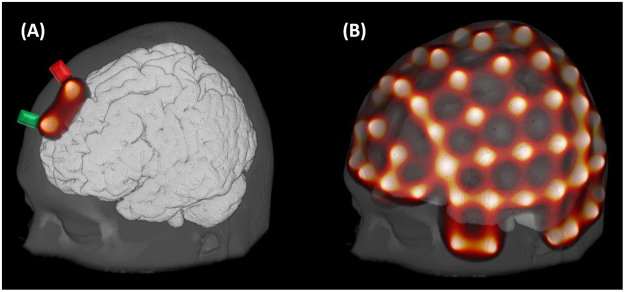
(**A**) Illustration of a single-channel photon transport simulation after sensitivity normalization. (**B**) Normalized sensitivity results for all channels considered based on sources and detectors positions depicted in Fig. 3B. In both cases, the color scale has been set from 10^−6^ (black) to 3*10^−3^ (white) and the head atlas was Colin27.

The sensitivity for each fNIRS channel (source-detector pair) was calculated as the voxel-wise product of the corrected photon fluence obtained for the source and the detector (adjoint field)^29^. This was normalized by the sum of sensitivity of all voxels, similarly as described by Brigadoi and Cooper, 2015^31^, so each voxel was represented by a percentage sensitivity in respect to the whole volume. Figure 4A depicts the normalized sensitivity result for channel formed by source Fz and detector AFz.

The normalized sensitivity was calculated for each fNIRS channel based on 38 sources and 36 detectors (Methods section) positioned on each head atlas (Methods section). 130 fNIRS channels have been formed by neighboring sources and detectors. The resulting normalized sensitivities for all channels on Colin27 left hemisphere are illustrated on Fig. 4B.

### Specificity and channels coordinates

After computing the normalized sensitivity (normSens), as described in Methods section, the brain sensitivity (brainSens) of a given channel (ch) with respect to a head atlas (h) is calculated. This is done by summing normSens from all voxels (i) classified as gray and white matter during tissue segmentation^27^, as follows:

1 $$brainSens(ch)=\sum _{i}^{brain}normSens(ch,i)$$

To better assess the influence of a given region of interest (i.e. set of voxels) inside the brain in respect to *brainSens(ch)*, we define “specificity” as follows:

2 $$Specificit{y}_{ROI}(ch)=100\times \sum _{j}^{ROI}\frac{normSens(ch,j)}{brainSens(ch)}$$

Equation 2 can be translated as a weighted mean of the voxels (j) within a given region of interest (ROI), in which the weight corresponds to the normalized sensitivity (normSens), normalized by the sensitivity to the brain (brainSens) to percentage. Thus, it provides the anatomical specificity of a channel (ch) to the region-of-interest (ROI).

Finally, we define the coordinates in the MNI space of a given channel as the weighted mean of the MNI coordinates of the voxels (k) within the brain that can be reached by a given channel. Similarly, the weight is the normalized sensitivity:

3 $$MN{I}_{x,y,z}(ch)=\sum _{k}^{brain}\frac{normSens(ch,k)\times MN{I}_{x,y,z}(k)}{brainSens(ch)}$$

According to equation (3), each fNIRS channel can be spatially represented with a set of MNI coordinates (x, y, z) in respect to the normalized sensitivity and brain sensitivity results obtained from the head atlas of interest (Colin27 or SPM12).

### Brain parcellation atlases

To assist on the experimental design of fNIRS in terms of definition of regions of interest, we considered five available parcellation atlases: (a) Automated Anatomical Labeling (AAL2)^32,33^, (b) Atlas of Intrinsic Connectivity of Homotopic Areas (AICHA)^34^, (c) Brodmann Areas^35^, (d) Jülich histological (cyto- and myelo-architectonic) atlas (Anatomy Toolbox)^36–38^, and (e) LONI Probabilistic Brain Atlas (LPBA40)^39^.

Each of these parcellation atlases is protected by copyright and provided by their developers “as is”: with no warranties and subject to change. Thus, we encourage readers to refer to the corresponding publications for details on the methodology.

**Figure 5:**
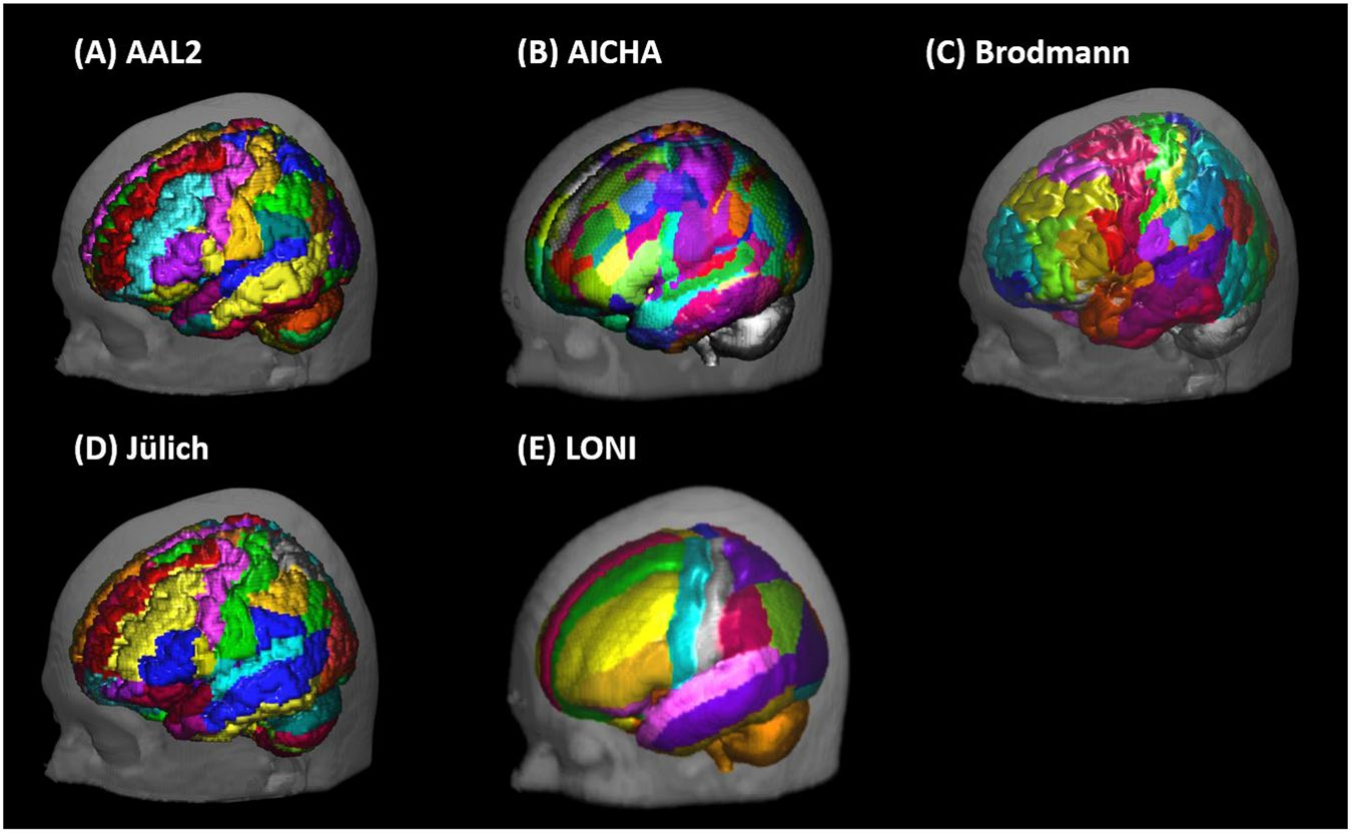
Illustration of brain parcellation atlases’ results incorporated in the toolbox: (**A**) Automated Anatomical Labeling (AAL2)^32,33^, (**B**) Atlas of Intrinsic Connectivity of Homotopic Areas (AICHA)^34^, (**C**) Brodmann^35^, (**D**) Jülich (SPM Anatomy Toolbox)^36–38^, (**E**) LONI Probabilistic Brain Atlas (LPBA40)^39^. (**A**,**C**,**D**) were overlaid on Colin27. And (**B**,**E**) overlaid on head atlas generated from tissue probability maps of SPM12.

Briefly, AAL was created from a parcellation of the high-resolution T1 volume of Colin27^16^ and its second and most recent version (AAL2) implemented a new parcellation of the orbitofrontal cortex. AICHA presents 192 functional regions as identified from resting-state data collected with fMRI from 281 individuals. Brodmann areas was acquired from MRIcron^17^, developed by Chris Rorden, with parcellation to the Colin27 atlas. Jülich histological atlas has been obtained from the SPM Anatomy Toolbox, based on post-mortem brains from ten subjects and normalized to Colin27 atlas. LPBA40 was created based on 40 healthy subjects and spatially normalized using different algorithms, from which we selected the SPM5 unified segmentation method^40^. The atlases are illustrated in Fig. 5.

As AAL2, Brodmann and Jülich atlases were registered to Colin27; we used it as reference head atlas for the anatomical labeling procedure. AICHA and LPBA40 were then considered within the head atlas generated from the tissue probability maps available in SPM12, as described in Methods section. The alignment of each parcellation atlas was compared with the head atlas of reference in SPM12. We used SPM12 function “Coregister: Estimate”^41^ to improve the alignment of Jülich parcellation atlas with the Colin27 head atlas that was obtained from MRIcron.

After alignment corrections between parcellation atlas and reference atlas, we computed the specificity of each landmark for a given channel according to the definition of equation 2, for which each ROI was defined as a landmark available in the parcellation method of interest. Remaining voxels of the reference atlas within the brain volume that did not present any overlap with the parcellation was classified as “Brain_Outside”. At the end of this, anatomical landmarks and respective specificity are assigned to each channel. For example, the results for channel formed by detector AFz and source Fz are presented in Table 2.

**Table 2:** Example of anatomical landmarks and specificity results for channel AFz-Fz and Brodmann parcellation method. Results related to coverage <10% have been omitted.

Anatomical landmark	Specificity (%)
9 - Dorsolateral prefrontal cortex	61.77
10 - Frontopolar area	20.26
8 - Includes Frontal eye fields	12.15

As the goal of the present method and toolbox is to retrieve channels as output of selected regions of interest, results have been grouped by landmarks, thus enabling look-up tables from anatomical landmarks of interest. An example is shown in Table 3 for landmark “Precentral_L” as defined in AAL2 and resulting channels information (source, detector and MNI coordinates).

**Table 3:** Example of channels with at least 10% specificity of “Precentral_L” (AAL2).

Source	Detector	Specificity (%)	Distance (mm)	X	Y	Z
C3	FC3	54.12	37	−50	−3	50
C3	C1	49.38	40	−42	−20	62
FC5	FC3	42.44	36	−55	12	34
FC1	C1	35.88	39	−26	−5	68
Cz	C1	31.89	39	−17	−20	74
FC1	FC3	21.10	36	−38	12	55
FC5	C5	16.66	33	−62	−3	23
CP1	C1	15.64	39	−27	−36	71

The inter-optode distances according to positions on Colin27 are provided. MNI spatial coordinates of each channel obtained based on rounded results from Equation 3.

Similar results as presented in Tables 2 and 3 have been stored in Matlab files (*.mat) for each parcellation method to be directly called from within fOLD (Toolbox section) to speed up the process and avoid repetitive results computation.

### Extension to 10-5 system

The method proposed so far is expected to provide a broad range of channels over the cortex and thus cover most regions that can be reached with fNIRS. However, as these channels are formed by sources and detectors placed on 10–10 international system, users interested in running EEG-fNIRS multi-modal measures would not be capable of using the toolbox, as EEG placement by standard is based on 10–20 and 10–10 international systems^12^.

**Figure 6:**
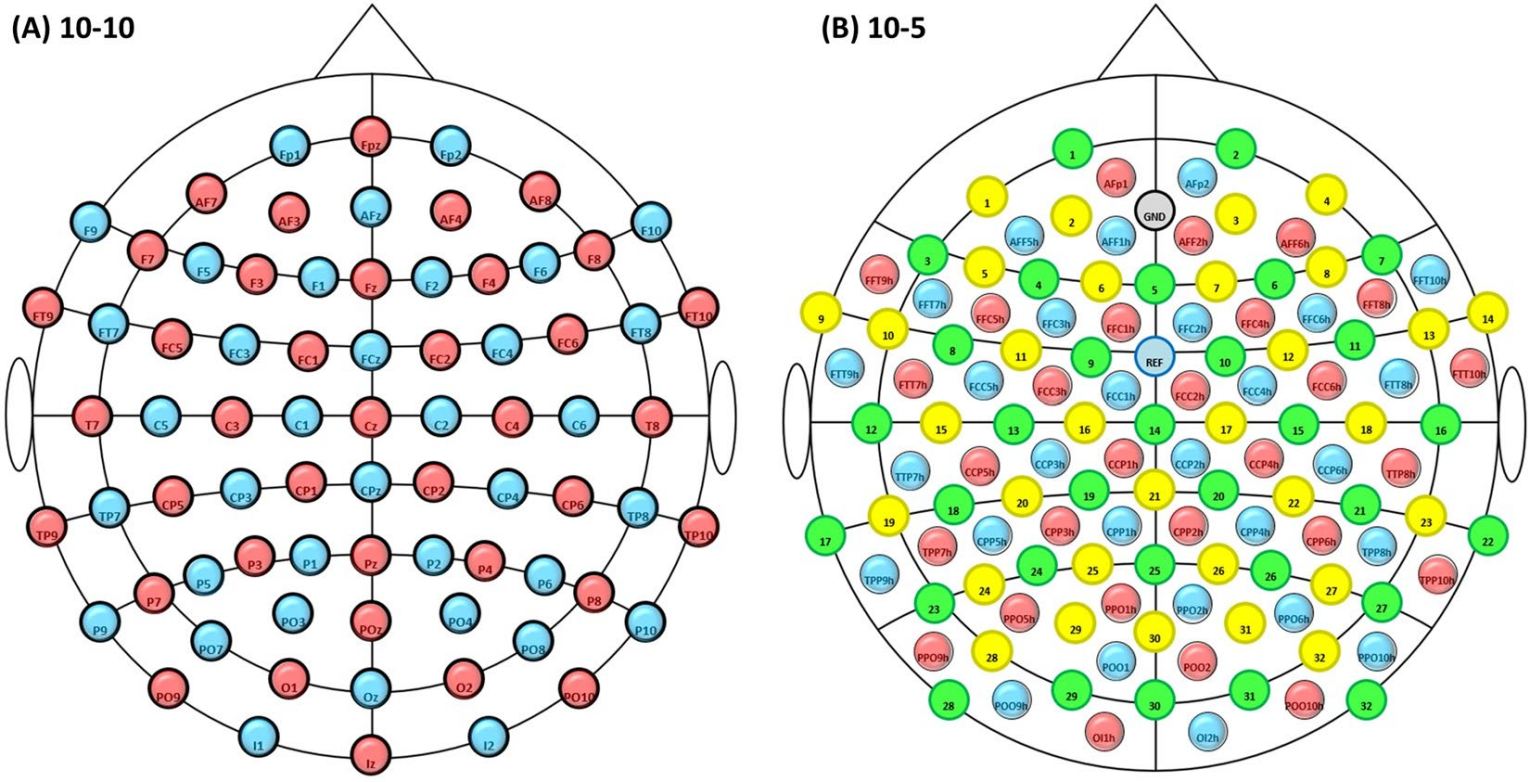
(**A**) Expansion of the method described for 10–10 international system to (**B**) the 10–5 system to allow for multi-modal measurements with EEG, either 32 or 64 electrodes. EEG and fNIRS positions are based on a layout accommodating 130 positions in total. EEG 1–32 electrodes positions are depicted in green, while the complimentary 33–64 are in yellow; fNIRS sources positions are in red and the detectors are in blue.

To extend the possible optodes positions to 10–5 international system positions regarding layouts available for EEG caps, we have considered as a reference a cap with 130 positions in total. With this new design, one has the possibility to use either 32 positions (Fig. 6A) or 64 positions for EEG electrodes (Fig. 6B), while the locations for fNIRS optodes do not overlap with the 10–10 system.

We have visually assigned sources and detectors to 10–5 system positions with the goal to maximize the number of possible channels considering adjacent optodes. This resulted on the layout illustrated in Fig. 6, which presents 28 sources positions and 28 detectors positions over the scalp. From these positions, we considered 89 possible channels in total.

Once the positions have been assigned and their coordinates have been retrieved for both head atlases (Methods section), we proceeded with the methods described in the Methods section. Therefore, we ran the photon transport simulations and computed the normalized sensitivity, ROIs specificity and channels coordinates, and obtained the anatomical landmarks results of each parcellation atlas.

The derived results were also stored in Matlab files included in the fOLD toolbox.

### Data availability

Authors can confirm that all relevant data are included in the paper and/or its supplementary material.

## Toolbox

**Figure 7:**
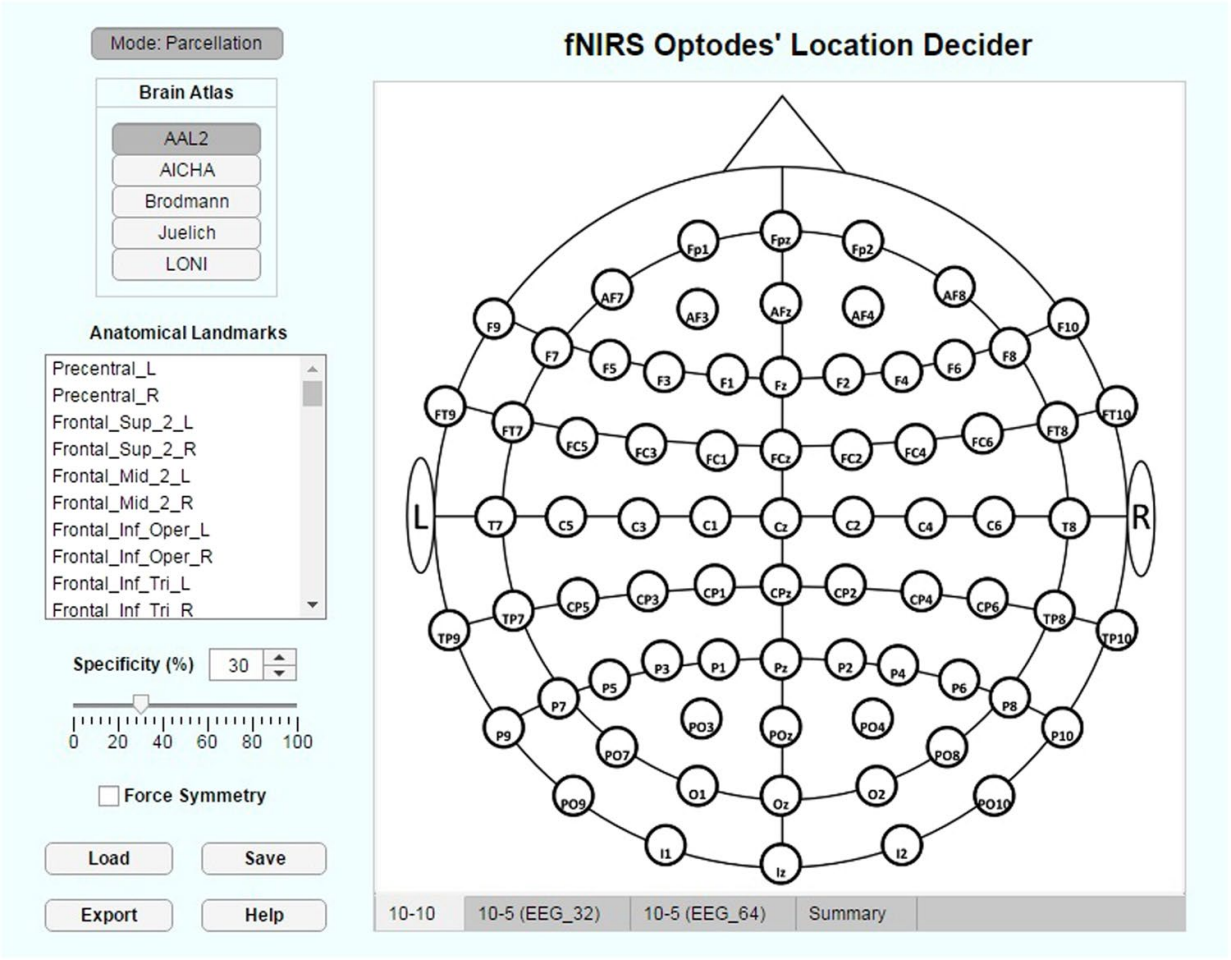
Graphical user interface of the fNIRS Optodes’ Location Decider (fOLD). Depicted is the blank 10–10 layout as displayed upon toolbox initialization.

From the methods described in Methods section and the derived results for both 10–10 and 10–5 extended positions for all brain parcellation methods considered and based on the two head atlases of reference, we developed, in Matlab2017a App Designer^42^, the toolbox “fNIRS Optodes’ Location Decider” (fOLD). The graphical user interface displayed upon software initialization is illustrated in Fig. 7.

On the top left corner, there is a list of parcellation atlases available (Methods section). The list of anatomical landmarks corresponds to the chosen atlas (in the figure, AAL2) that can be reached by any of the fNIRS channels resulted from the methods described in Methods section.

Under the anatomical landmarks list, there is a specificity threshold that can be set either numerically or with a sliding bar to define the lower specificity limit to the selected landmarks that a channel must present to be included in the fNIRS optodes arrangement (Methods section). The “force symmetry” checkbox allows the user to force the algorithm of optode positions selection to always generate symmetric locations (in terms of placed optodes) between the left and the right hemisphere.

The button “Save” allows the user to store the current arrangement settings to load in a posterior session by clicking on “Load”. “Export” button allows saving the most relevant information on the current positions as text and Excel files (*.xls). “Help” will display yellow text boxes with complimentary information about important features.

On the main panel, the chosen layout (10–10, 10–5 EEG_32 or 10–5 EEG_64 – Methods section) will be depicted and automatically populated with sources (red) and detectors (blue) optodes following chosen mode, parcellation method, anatomical landmarks and minimum specificity threshold.

**Figure 8:**
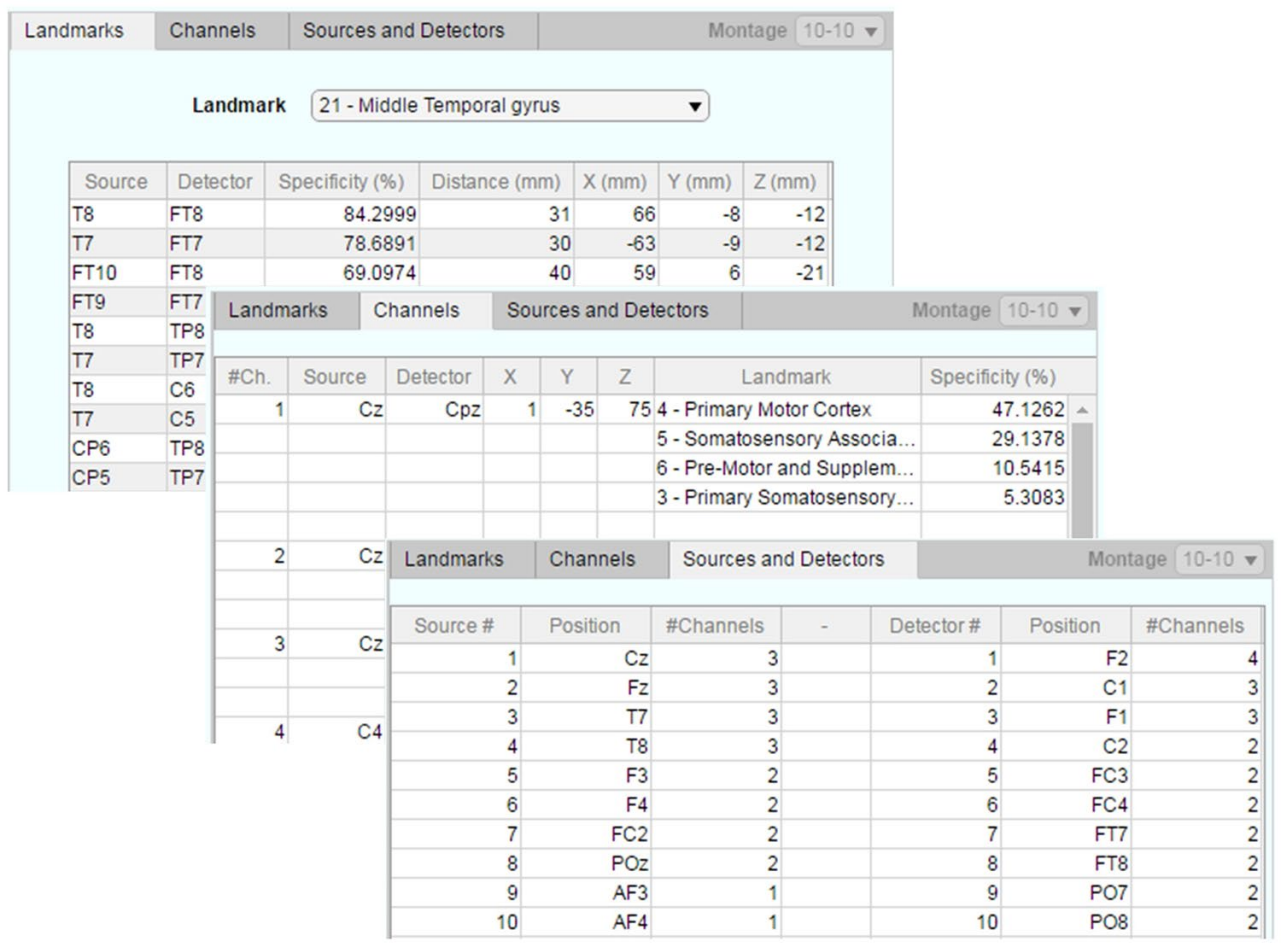
Illustration of further tabs available under “Summary”. ‘Landmarks’ (top), ‘Channels’ (center) and ‘Sources and Detectors’ (bottom), each of them presenting tables with summarized information about the fNIRS optode layout designed.

By clicking on the “Summary” tab, the user can access most relevant information about the created optodes arrangement. As illustrated in Fig. 8, the information is summarized in three different sub-tabs: (i) Landmarks, (ii) Channels, and (iii) Sources and Detectors. The first presents information of all channels generated for a given landmark of interest, similar as shown in Table 3. ‘Channels’ will display a table with formed channels and list of landmarks covered by each of them, similar as in Table 2. The last tab (‘Sources and Detectors’) presents a list of sources and detectors required and sorted by the number of channels formed.

In addition to the choice of anatomical landmarks based on parcellation atlases, one can also switch the working mode to “Image Mask”, by clicking on “Mode: Parcellation”. This will pop up a message dialog to the user informing the switching to “Image Mask” and, if accepted to proceed, the user will be prompted to choose a NIfTI or ANALYZE file.

The selected image file will be loaded in the toolbox, resampled to 2 × 2 × 2 mm^3^, and the toolbox will look for any overlap of its voxels presenting values greater than 0 with those of each fNIRS channel extent. For each channel, the specificity to the loaded region of interest will be calculated (Equation 3) and the optodes related to channels surviving the minimum specificity threshold will be displayed in the main panel.

**Figure 9:**
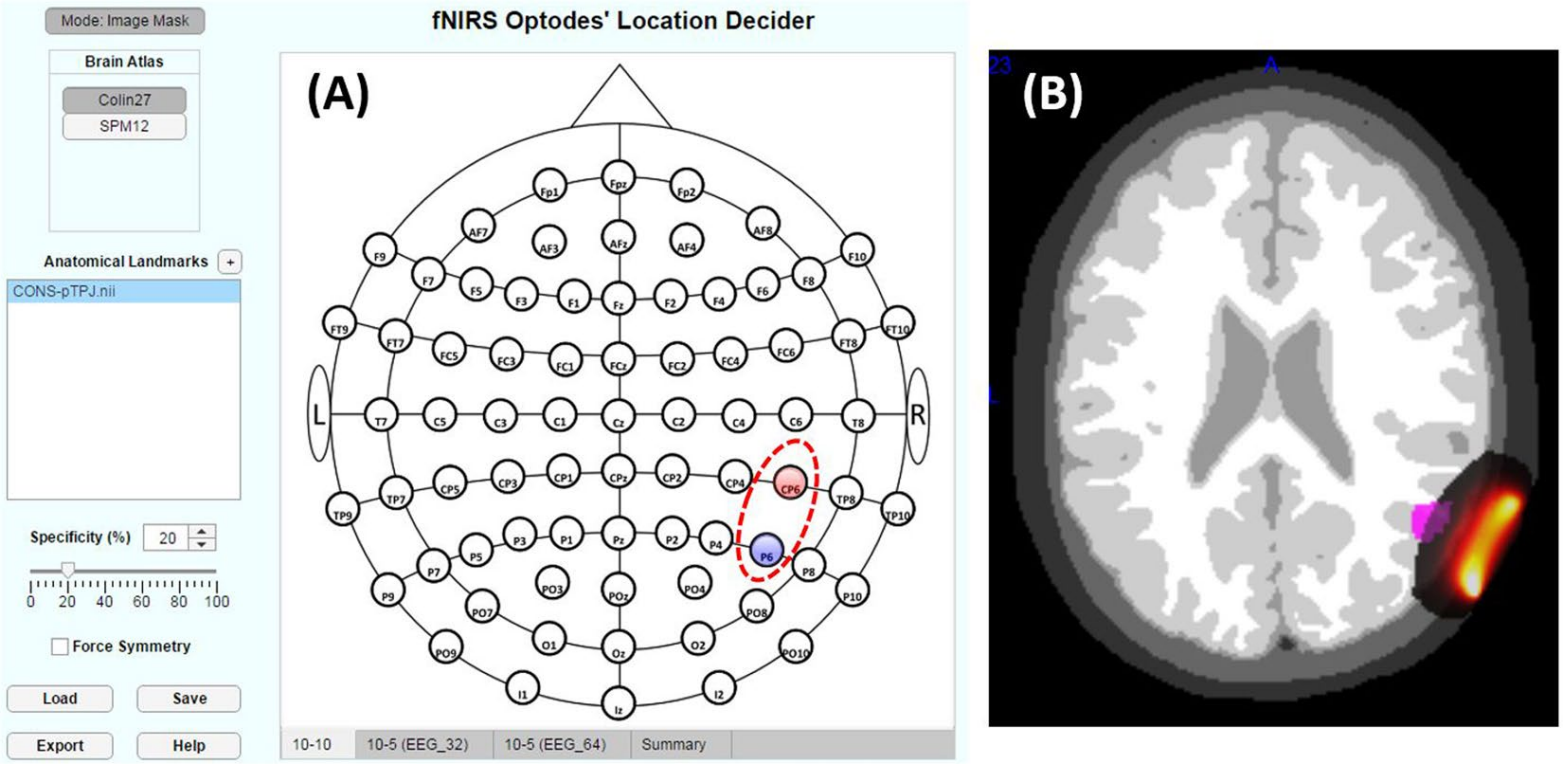
Representative NIfTI file for posterior temporal-parietal junction (pTPJ) as obtained from Archive of Neuroimaging Meta-Analyses (ANIMA)^50^ and published by Bzdok *et al*.^53^. was loaded in the fOLD toolbox in the ‘Image Mask’ Mode, (**A**) resulting on channel formed by positions CP6 and P6. (**B**) Overlay of pTPJ (in violet) on Colin27 segmented atlas with an additional overlay of sensitivity for channel CP6-P6.

Figure 9 illustrates an example of NIfTI file representing the posterior temporal- parietal junction (pTPJ) loaded in “Mode: Image Mask” and resulted on a channel CP6-P6 given the set minimum specificity threshold of 20%. The same figure also illustrates an axial view of the Colin27 segmented head with overlays of the pTPJ NIfTI file and the normalized sensitivity (Methods section) of the channel CP6-P6, showing their overlap. It is interesting to note that the positions yielding a greater spatial specificity to the pTPJ did not coincide with the usual choice of international positions labeled as “TP” (temporal-parietal).

In “Mode: Image Mask”, the options available under “Brain Atlas” are not related to the parcellation atlases anymore, but rather to the head segmented atlases Colin27 and SPM12 (Methods section). Also, next to “Anatomical Landmarks”, the user can load further images to be included in the landmarks list for more robust creation of fNIRS optodes arrangement from NIfTI or ANALYZE files.

## Discussion and Conclusions

The current study presented a toolbox to facilitate the selection of fNIRS optode scalp positions based on the overlapping of regions of interest within the brain and the simulated photon transport from optodes positioned in 130 positions on the cap.

The toolbox incorporates the overlapping results obtained from the anatomical landmarks from five different parcellation methods (Methods section), while it also allows the user to load an image file of interest to be used as mask for the positions definition (Toolbox section and Fig. 9).

Output results of the toolbox are presented on the reference cap layout of interest (10–10, 10–5_EEG-32 or 10–5_EEG-64 – Methods section), as well as in summarizing tables. The tables provide summarized information on anatomical landmarks’ (ROI) specificity (equation 2), channels’ MNI coordinates (equation 3) and number of channels per optode (Fig. 8). The summary information can also be exported to Excel sheets (*.xls) and to text files (*.txt), as described in Toolbox section.

The current version presents following limitations: (I) the resulting channels can only be formed either for positions based on 10–10 or 10–5 extent, i.e. one cannot combine them into a single layout; (II) high-density arrangements for 3D diffuse optical tomography^43,44^ cannot be automatically generated from within fOLD; (III) the normalized sensitivity is separately calculated on a channel basis and does not accumulate for near channels with eventual overlapping photon transport. These first three limitations mainly concern the possibility of extending the presented approach to appreciate the potential for tomography studies, while the current version of fOLD rather focuses on topography (i.e. in the absence of major overlap of sensitivity profiles of photon transport). Nevertheless, we believe that one can potentially identify the most relevant positions from within fOLD and extend its output to a higher-density of optodes by placing more channels around the retrieved topographical positions.

Additionally, further limitations concern the implemented methodology: (A) the photon transport simulations have been generated with two distinct head atlases (Colin27 and SPM12), whose tissues’ geometries (e.g. scalp and skull thickness) may significantly differ from other adult individuals^27^; (B) our results should only be valid for adults, as we did not consider head atlases for children or infants^45^; (C) there is seemingly no gold standard on values for tissues’ optical properties, as recent studies based on photon transport simulations applied different values from one another^27,30,31,46^; (D) the fiducials points for each head atlas have been visually identified, while it has been shown that the visual identification of inion may be ambiguous^12,47^; (E) different tissue segmentation methods available may yield different thickness results^48^, although SPM12 has not been assessed yet; (F) the results provided as output of the toolbox are restricted to the 10–10/10–5 international systems positions^12^.

Also, due to the predefined potential positions for the optodes as based on the international systems (10–10 and 10–5), the resulting inter-optode distance is not constant for the resulting channels. Nevertheless, to avoid too long distances that could not provide measurements with a proper signal-to-noise ratio, we only considered channels formed by neighboring optical positions on 10–10 or 10–5. This yielded a median inter-optode distance of 3.60 cm, while the upper quartile is 3.98 cm and the lower quartile is 3.13 cm. There are only five possible channels that exceed 4.50 cm (two from 10–10 and three from 10–5). Given proper headgear preparation and detected signal amplification, we envision that fNIRS users can obtain reasonable signal-to-noise ratio with the channels output of the toolbox.

In the context of previously proposed methods to address the definition of optodes position for fNIRS to target a set of brain regions-of-interest^9–11^, the main feature differentiating our method and developed toolbox is its simplification and consequent accessibility. The simplification relies on the fact that fOLD does not require digitalization of optodes positions nor individual structural scans, and the results of photon transport simulations have been computed offline and are readily available within the toolbox. Additionally, the accessibility of the method could be achieved by considering the 10–10 and 10–5 international systems as references for optodes arrangement and by relying on head atlases as templates for photon transport simulations. Thus, fOLD can be accessed and used by groups with different levels of expertise and background, while it does not require additional hardware (digitizer and MR scanner), nor further photon transport simulations. Nevertheless, the results are obtained independently of anatomic variances between-subjects, and it requires the optodes to be precisely placed in accordance to the international systems^12^. In agreement with the conclusions presented by Machado *et al*.^9^, we believe that these simplifications are acceptable for fNIRS non-clinical applications. Also, Wijeakumar *et al*.^10^ had reported significant correlation of the results obtained for subject-specific and head atlas in adults. Finally, using head atlases as templates facilitates both the application of brain parcellation atlases (Methods section) and the fNIRS positions definition based on meta-analyses (Toolbox section), as the latter was also envisioned by Wijeakumar *et al*.^10^ as a future approach to identify ROIs.

In future versions of fOLD, we intend to include: (1) the possibility to display lines between sources and detector positions to illustrate the formed channels; (2) an optimization algorithm to find the best positions for a given number of sources and detectors; (3) a threshold for the source-detector distance; and (4) an additional threshold metric based on the brain sensitivity (Equation 1) to also assist on the choice of channels that present a higher sensitivity to the brain. We envision that the brain sensitivity information could provide a better prediction on the signal-to-noise ratio of a given channel following removal of extracortical components from its time series. Channels with lower brain sensitivity have a lower portion of cortical components and thus one can expect a lower signal-to-noise ratio in respect to the extracortical signals. Nevertheless, while this has not yet been incorporated into fOLD, it is provided within the supplementary datasets. Also, given that one is capable of properly retrieving the cortical component from the measured channel, for example by including short-distance channels to measure the extracortical changes^49^, we expect the currently provided threshold of specificity to best represent the portion of region-of-interest covered, regardless the sensitivity to the brain.

To conclude, we consider that the presented toolbox is a first-order approach to bring the achieved advancements within fMRI concerning parcellation methods (Methods section) and meta-analyses^50–52^ to the fNIRS optode positions selection. While it is known that the fNIRS spatial resolution is lower than fMRI, it is possible to increase the precision of hypothesis-driven experimental design by finding the overlapping regions of interest with the simulated fNIRS sensitivity profiles, as illustrated in Fig. 9 based on the meta-analysis of posterior temporal parietal junction^53^. We envision that the toolbox shall not only benefit the design of fNIRS experiments and optode positions but also improve the discussion of fNIRS results in face of *a priori* expectation of channels to present a greater anatomical specificity to a region of interest. Finally, the proposed MNI coordinates (Equation 3) calculated from the weighted mean, based on a given channel’s normalized sensitivity, may also further improve results comparison with fMRI and enable fNIRS meta-analyses.

The fOLD toolbox is provided as standalone version for Windows (*.exe) or as App package for Matlab. The App package is compatible with Windows, Mac or Ubutu, but currently only for Matlab2017a. The most recent version of fOLD is available in the following GitHub public repository: https://github.com/nirx/fOLD-public.

## Electronic supplementary material

Supplementary Information

Dataset A1

Dataset A2
