# Supplementary material for: fNIRS Optodes’ Location Decider (fOLD): a toolbox for probe arrangement guided by brain regions-of-interest
Source: Sci Rep. 2018 Feb 20;8:3341. doi: 10.1038/s41598-018-21716-z (PMC5820343; doi:10.1038/s41598-018-21716-z)
Supplement: Supplementary file 1 — Supplementary Information [file 41598_2018_21716_MOESM1_ESM.pdf]

Supplementary Information

fNIRS Optodes' Location Decider (fOLD): a toolbox for probe arrangement guided by brain regions-of-interest

Guilherme Augusto Zimeo Morais*, Joana Bisol Balardin, João Ricardo Sato

*Corresponding author - guilherme.zimeo@nirx.de

Supplementary datasets ("Dataset A1" and "Dataset A2") are included along the manuscript.

Each uploaded dataset corresponds to a different standardized reference for fNIRS optodes positioning (Dataset A1: 10-10 and Dataset A2: 10-5).

Datasets are Excel files with relevant results obtained for the parcellation atlases currently incorporated in the fOLD toolbox. For each parcellation, three sheets are presented:

1. Summary of landmarks derived from a given parcellation method (AAL2, AICHA, Brodmann, Jülich or LONI). The sheet name corresponds to the parcellation method (e.g. AAL2).

2. Detailed information of fNIRS channels with resulting specificity greater than 1% to each landmark listed in the first sheet. Included are sources and detectors positions based on the reference international system (10-10 or 10-5), resulting specificity (%), resulting inter-optode distance (mm) and channel's coordinates in MNI space (mm). The sheet name comprehends the numbering of the parcellation atlas followed by "_Lnd". For example, Brodmann (atlas #3) results are presented within sheet "3_Lnd".

3. Relevant information for each fNIRS channel considered within international system of reference (10-10 or 10-5): (a) source and detector positions, (b) resulting inter-optode distance (mm), (c) brain sensitivity (%), (d) MNI coordinates (mm), (e) list of covered landmarks according to the chosen parcellation atlas, and (f) resulting specificity (%). Shown are only landmarks that the fNIRS channel presented at least 1% of specificity. The sheet name comprehends the numbering of the parcellation atlas followed by "_Chn". For example, Juelich (atlas #4) results are presented within sheet "4_Chn".

Please refer to the original manuscript for detailed information on the head and parcellation atlases considered, definition of fNIRS positions as well as calculation of results presented in the supplementary datasets. For example, brain sensitivity is described in Equation 1, ROI specificity in Equation 2, and MNI space coordinates for a given channel is in Equation 3.

Frequently Asked Questions (FAQ)

fNIRS Optodes' Location Decider (fOLD): a toolbox for probe arrangement guided by brain regions-of-interest

Guilherme Augusto Zimeo Morais*, Joana Bisol Balardin, João Ricardo Sato

*Corresponding author - guilherme.zimeo@nirx.de

1) Force Symmetry

Why is it necessary to use the "force symmetry" option?
Why do symmetrical anatomical landmarks yield different layout results?

The observed asymmetry occurs because the anatomical landmarks available in these parcellation atlases are not symmetrical. If one overlays e.g. the AAL2 to the Colin27 atlas (ch2.nii.gz) in MRIcron, one can clearly observe this. For example, the coordinates (-14,12,60) mm^3 corresponds to Frontal_Sup_2_L, while the coordinates (14,12,60) mm^3 are rather labeled as Supp_Motor_Area_R

2) Summary List

Why some possible channels do not appear in the summary list when they can physically exist?

An example would be AAL2, frontal mid 2 R, specificity 23 %, channel between F2 and FC2.

The summary list presents only the channels whose anatomical specificity to a given region surpassed the user-defined threshold. Therefore, if one uses the default threshold (30%) and selects the frontal mid 2 R with AAL2, this will not be listed there. However, if one decreases the threshold to 20%, the summary list will be automatically updated and the channel will be listed.

3) Incompatibility Issues

Please note that fOLD is available either as executable (Windows compatible) or as App to be installed in Matlab (Windows, Unix or Mac). However, the latter option is only compatible with the currently most recent version of Matlab (2017a), as this is the framework used for fOLD development.

If you face on of the following issues, this might be related to incompatible Matlab versions.
Therefore, we recommend you attempting to update Matlab before installing and running fOLD (App):

Following error occurs when attempting to run fOLD App within Matlab2016a:

Error using uitable

While setting the 'Parent' property of 'Table':

Functionality not supported with figures created with the uifigure function. For more information, see Graphics Support in App Designer.

Following error occurs when attempting to run fOLD App within Matlab2013a:

Error using nargout
Function fOLD does not exist.
Error in fOLDApp/startApp (line 57)
if nargout(@fOLD) == 0
Error in fOLDApp (line 37)
startApp(obj)
Error in appinstall.internal.runapp>execute (line 75)
out = evalin('caller', [script ';']);
Error in appinstall.internal.runapp>runapp13a (line 57)
outobj = execute(fullfile(appinstalldir, [wrapperfile 'App.m']));
Error in appinstall.internal.runapp>runcorrectversion (line 35)
appobj = runapp13a(appinstalldir);
Error in appinstall.internal.runapp (line 17)
out = runcorrectversion(appmetadata, appentrypoint, appinstalldir);
